# All-inside arthroscopic procedures for chronic lateral ankle instability: evidence-based clinical practice guidelines

**DOI:** 10.1093/bmb/ldaf001

**Published:** 2025-04-04

**Authors:** Shi-Ming Feng, Nicola Maffulli, C Niek van Dijk, Hai-Lin Xu, Kai Fehske, Christian Plaass, Francesco Oliva, Thomas Karius, Shun-Hong Gao, Wei Xu, Lu Bai, Run-Lai Song, Yue-Feng Hao, Hui Zhang, Yang-Bo Xu, Ning Zhang, Lei Lou, Tian-Tian Ren, Guo-Dong Wang, Qi Li, Jian-Hua Wu, Yong-Zhan Zhu, Gang Yin, Tong-Fu Wang, Jian-Zhong Qin, Amol Saxena, Chao Ma

**Affiliations:** Sports Medicine Department, Xuzhou Central Hospital, Xuzhou Clinical College of Xuzhou Medical University, NO.199 Jiefang South Road, Quanshan district, Xuzhou 221009, PR China; Department of Orthopaedics and Traumatology, University La Sapienza, Faculty of Medicine and Psychology, Via di Grottarossa, 00189, Rome, Italy; Guy Hilton Research Centre, School of Pharmacy and Bioengineering, Keele University, Thornburrow Drive, Stoke-on-Trent ST4 7QB, United Kingdom; Centre for Sports and Exercise Medicine, Barts and The London School of Medicine and Dentistry, Mile End Hospital, 275 Bancroft Road, London E1 4DG, United Kingdom; Department of Orthopedic Surgery, University of Amsterdam, Meibergdreef 9, Amsterdam, 1081 HV, The Netherlands; Ankle Unit, FIFA Medical Centre of Excellence Ripoll-DePrado Sport Clinic, C. de Almagro, 34, Chamberí, Madrid 28010, Spain; Ankle Unit, FIFA Medical Centre of Excellence Clínica do Dragão, Estádio Dragão Entrada Nascente, Porto 4350-415, Portugal; Casa di Cura, San Rossore, Viale delle Cascine, 152/f, Pisa 56122, Italy; Department of Trauma and Orthopedic, People’s Hospital, Peking University, No. 11 Xizhimen South Street, Xicheng District, Beijing 100044, PR China; Department of Trauma Surgery, University Hospital Wuerzburg, Josef-Schneider-Straße 2, Wuerzburg 97080, Germany; Department of Orthopedic and Trauma Surgery, Johanniter Waldkrankenhaus Bonn, Waldstraße 73, Bonn 53177, Germany; Department for Foot and Ankle surgery, DIAKOVERE Annastift, Orthopedic Clinic of the Hannover Medical School, Anna-von-Borries Strasse 1-7, Hannover 30625, Germany; Department of Sports Traumatology, Universita’ Telematica San Raffaele, Via di Val Cannuta, 247 Roma, Italy; Department of Orthopedic and Trauma Surgery, Johanniter Waldkrankenhaus Bonn, Waldstraße 73, Bonn 53177, Germany; Orthopaedic Department, The Second Hospital of Tangshan, No. 21, North Jianshe Road, Lubei District, Tangshan 063000, PR China; Department of Orthopaedics, The Second Affiliated Hospital of Soochow University, No. 1055, Sanxiang Road, Gusu District, Suzhou 215004, PR China; Department of Orthopaedics, Peking University Shenzhen Hospital, No. 1120, Lianhua Road, Futian District, Shenzhen 518035, PR China; Orthopedic Medical Center, Yibin Second People's Hospital, No. 96 Beida Street, Cuiping District, YiBin 644000, China; Orthopedics and Sports Medicine Center, Suzhou Municipal Hospital, Nanjing Medical University Affiliated Suzhou Hospital, No. 242, Guangji Road, Gusu District, Suzhou 215008, PR China; Department of Orthopedics and Orthopedic Research Institute, West China Hospital, Sichuan University, No. 37, Guoxue Lane, Wuhou District, Chengdu 610041, China; Department of Bone and Joint Surgery, Affiliated Hospital of Southwest Medical University, No. 8, Section 2, Kangcheng Road, Jiangyang District, Luzhou 646099, PR China; Department of Foot and Ankle Surgery, The Second Hospital of Shandong University, 247 Beiyuan Street, Tianqiao District, Jinan 250033, PR China; Department of Orthopaedics, Luoyang Orthopedic-Traumatological Hospital of Henan Province, No. 82, Qiming South Road, Chanhe Hui District, Luoyang 471002, PR China; Department of Orthopaedics, The First Affiliated Hospital of Ningbo University, No. 59 Liuting Street, Haishu District, Ningbo 315000, PR China; Department of Orthopaedics, Affiliated Hospital of Jining Medical University, No. 89 Guhuai Road, Rencheng District, Jining City 272000, PR China; Sports Medicine Center, West China Hospital, Sichuan University, No. 37, Guoxue Lane, Wuhou District, Chengdu 610041, China; Department of Trauma Orthopedics, The Affiliated Hospital of Guizhou Medical University, No. 28, Guiyi Street, Yunyan District, Guiyang 550004, China; Department of Orthopedics, Foshan Hospital of Traditional Chinese Medicine, No. 6, Qinren Road, Chancheng District, Foshan 528000, China; Department of Orthopedics, Binzhou Medical University Hospital, No. 661, Huanghe 2nd Road, Bincheng District, Binzhou 256603, China; Department of Sports Medicine and Arthroscopy, Tianjin Hospital of Tianjin University, No. 406, Jiefang South Road, Hexi District, Tianjin 300211, China; Department of Hand and Foot, The Second Affiliated Hospital of Soochow University, No. 1055, Sanxiang Road, Gusu District, Suzhou 215004, PR China; Department of Sports Medicine, Sutter-PAMF, 795 El Camino Real, Palo Alto, CA 94301, United States; Sports Medicine Department, Xuzhou Central Hospital, Xuzhou Clinical College of Xuzhou Medical University, NO.199 Jiefang South Road, Quanshan district, Xuzhou 221009, PR China

**Keywords:** chronic lateral ankle instability, arthroscopic management, guidelines

## Abstract

**Background:**

All-inside arthroscopic procedures are now frequently employed to manage chronic lateral ankle instability (CLAI) with satisfactory functional outcomes. Currently, no evidence-based guidelines exist for all-inside arthroscopic procedures for CLAI. Many surgical decisions remain uncertain and challenging.

**Sources of data:**

Published scientific literature in PubMed, MEDLINE, Web of Science, EMBASE, and Cochrane databases.

**Areas of agreement:**

All-inside arthroscopic repair and reconstruction procedures are reliable treatments for CLAI.

**Areas of controversy:**

The all-inside arthroscopic procedures for CLAI present significant challenges, particularly in the following aspects:

- surgical indications
- portal selection
- the number of inserted anchors
- suture configurations
- anterior talofibular ligament repair procedures
- reconstruction strategies
- remnant preservation
- treatment of osteochondral lesions of the talus
- postoperative rehabilitation protocols.

**Growing points:**

Given the lack of guidelines for the all-inside arthroscopic procedures for CLAI, this evidence-based clinical practice guideline provides 11 recommendations to address the controversy.

**Areas timely for developing research:**

In patients with CLAI undergoing all-inside arthroscopic procedures, comparative studies are urgently needed to establish the optimal timing for weight-bearing, as well as return to work and sports.

## Introduction

Chronic lateral ankle instability (CLAI) is common and can cause significant limitations in daily activities. CLAI is defined as repeated giving way of the ankle joint, resulting in instability, pain, and decreased function. While conservative measures such as physical therapy and bracing can be effective, many individuals with CLAI will require surgical intervention. Over the past few decades, with the development of arthroscopic technology, ankle ligament repair assisted by arthroscopy has become increasingly popular. The all-inside arthroscopic ligament repair technique is widely used, as it enables simultaneous intra-articular lesion management while minimizing incisions and ensuring good clinical outcomes [1–11]. However, despite its advantages, there remain critical gaps in understanding the optimal application of this procedure. These include the appropriate surgical indications, the selection of techniques, postoperative rehabilitation protocols, and strategies to prevent complications. Given the lack of unified standards in the arthroscopic management of CLAI, significant variability exists in the approaches adopted by different practitioners and institutions. To address these gaps, this guideline aims to provide evidence-based recommendations for the arthroscopic management of CLAI.

## Materials and methods

### Purposes of developing clinical practice guidelines

The current clinical practice guidelines serve as a valuable reference for orthopedic surgeons attending to CLAI patients undergoing all-inside arthroscopic procedures. These guidelines are designed not only to offer practical assistance to professionals but also to provide standardized medical information for orthopedic surgeons who are responsible for the surgical management of CLAI patients.

### Development process of the clinical practice guidelines

#### Selection of the key questions

Twenty orthopedic surgeons were consulted to provide their expertise and insights on guidelines concerning all-inside arthroscopic procedures for CLAI. They reviewed and discussed the clinical guidelines developed by the Chinese Society of Sports Medicine for the surgical management of CLAI, selecting key questions for the clinical practice guidelines for all-inside arthroscopic procedures. Key questions (Table 1) were selected with consideration given to the following areas: indications for all-inside arthroscopic procedures, portals, surgical techniques, postoperative rehabilitation protocols, and guidelines for returning to work and sports.

**Table 1:** Key questions for all-inside arthroscopic procedures for CLAI

Categories	Key question
Indications	What are the indications for all-inside arthroscopic procedures for CLAI?
Portals	What portals should be used?
Surgical techniques	How to address the OCL combined with CLAI?
	One versus two anchors—which is better?
	What is the optimal anchor insertion angle?
	What suture configuration should be used?
	Is it necessary to preserve the stump when performing the anatomic reconstruction?
	How to deal with an unstable os subfibulare?
	Anatomic reconstruction, Broström or Broström–Gould repair—which is better?
Postoperative rehabilitation protocols	When to start range of motion and weight-bearing?
Returning to work and sports	When to return to work and sports?

#### Literature search strategy

The selection of keywords and the formulation of search strategies were determined through discussions between the authors responsible for each key question and the expert methodologist.

**Figure 1:**
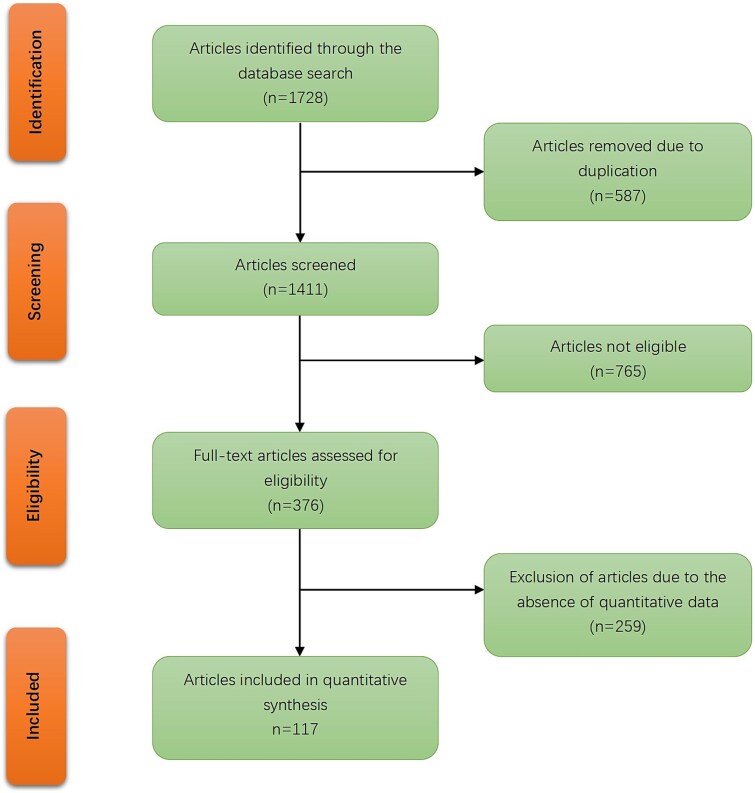
Flowchart of the literature search.

A comprehensive literature search was performed using specific keywords and strategies to identify articles published between January 1980, when arthroscopy became more widely adopted, and September 2024. The search covered the PubMed, MEDLINE, Web of Science, EMBASE, and Cochrane databases. The inclusion criteria for this search were original articles, reviews, and abstracts which involved both adults and children. Studies were excluded if they were editorials, letters, lecture notes, or case reports. Initially, articles were screened based on their titles and abstracts. Full texts of the selected articles were then reviewed for eligibility. Two members of the working group independently reviewed articles for each key question according to the inclusion criteria, resolving any disagreements through discussion (Fig. 1).

### Evidence grading

To summarize the findings of the included studies, the researchers evaluated each study’s design and potential for bias using the Cochrane Handbook for Systematic Reviews of Interventions for Assessing the Risk of Bias for articles reporting randomized studies and the Newcastle-Ottawa Scale for articles reporting nonrandomized studies. This approach ensured a comprehensive assessment of research quality across different study designs. The strength of evidence was evaluated through the Grading of Recommendations Assessment, Development, and Evaluation system. The level of evidence was classified as follows: High, indicating that further research is unlikely to alter the confidence in the estimated effect; Moderate, where further research could significantly impact confidence and potentially change the estimate; Low, suggesting a high likelihood that further research will notably affect the certainty and potentially revise the estimate; and Very Low, where predicting the effect is not feasible.

#### The strength of the recommendation

The strength of the recommendation was categorized as Strong, meaning that the intervention is highly recommended for most clinical situations given its significant benefits relative to risks and robust evidence base; Weak, indicating that the intervention should be applied selectively or under specific conditions, as its effectiveness may vary based on clinical context or patient values; and Expert Consensus, where, despite limited clinical evidence, the intervention is advised based on its benefits, risks, level of evidence, patient values, preferences, and available resources, with the decision guided by clinical experience and expert opinion.

During the literature search, articles addressing the key questions were prioritized. If there was insufficient evidence because of high heterogeneity among the selected articles or a lack of relevant articles, other clinical practice guidelines and review articles related to the questions were consulted to draft the recommendations. In such cases, the strength of the recommendation was based on expert consensus derived from the systematic review of the available literature.

#### Review and approval

Based on the findings, a preliminary draft of the guideline statement detailing the recommendations was produced. The recommendation grading process was reviewed and approved if more than 70% of the votes were in favor. In the first round of voting, participants used a 5-point Likert scale via email to indicate their level of agreement (completely agree, generally agree, partially agree, generally disagree, and completely disagree). A recommendation was accepted if at least 70% of the votes were either “completely agree” or “generally agree.” As a result, eight recommendations were approved, while three were not supported. In the second round of voting, consensus was reached on three revised recommendations. Ultimately, 11 recommendations were approved and adopted.

## Results and discussion

### What are the indications for all-inside arthroscopic procedures for CLAI?

#### Recommendation

All-inside arthroscopic management is indicated when (i) Patients experience functional impairments (such as pain, recurrent ankle sprains, or giving way) despite more than 6 months of conservative management; (ii) A positive anterior drawer test or talar tilt test, along with imaging (MRI, stress radiography or stress ultrasound) confirms CLAI (strength of recommendation: expert consensus; level of evidence: not applicable).

#### Statement

Currently, no randomized controlled or observational studies have specifically investigated the indications for all-inside arthroscopic procedures for CLAI. Therefore, the indications for this procedure were inferred from studies involving patients who have undergone all-inside arthroscopic treatment. The all-inside arthroscopic procedure is most commonly indicated for CLAI patients who exhibit symptoms such as ankle instability (giving way), recurrent sprains, or persistent pain after undergoing conservative treatment [1–11]. While some studies recommend a duration beyond 3 months [4,12–18], 6 months is the most widely accepted timeframe [8,19–31]. Preoperative physical examination results, such as a positive anterior drawer test or talar tilt test, are essential indicators to plan an all-inside arthroscopic procedure [11,14,17,22,25,27,29,32,33]. In addition, preoperative imaging is routinely conducted to ensure that surgical indications are supported by corresponding imaging findings. MRI, stress radiography, and stress ultrasound are the most commonly used imaging modalities [11,12,16–19,22,25,32,34–43].

### What portals should be used?

#### Recommendation

The anteromedial, anterolateral, accessory anterolateral and sinus tarsi portals are the most commonly used approaches (strength of recommendation: expert consensus; level of evidence: not applicable).

#### Statement

Currently, no randomized controlled or observational studies have evaluated whether the choice of portals in all-inside arthroscopic surgery affects surgical outcomes. Based on the available evidence, the anteromedial and anterolateral portals are the most commonly used in arthroscopic procedures [44–51]. These standard portals provide optimal visualization and facilitate the management of intra-articular ankle pathologies [52–56]. However, a standard anteromedial portal may not provide complete visualization of the tip of the lateral malleolus, suggesting that it might be inadequate to thoroughly observe the anterior talofibular ligament (ATFL) attachment site [57]. However, producing the portals with the ankle in dorsiflexion without distraction may allow better inspection of the lateral gutter and ATFL. Additionally, a growing number of surgeons use an accessory anterolateral portal, located 1.5 cm anterior to the distal tip of the fibula, as a working portal to facilitate ligament repair procedures [58,59]. During ligament reconstruction surgery, the sinus tarsi portal is typically positioned at the intersection of the posterior edge of the extensor retinaculum and the superior edge of the peroneus brevis tendon, while the retromalleolar portal is usually placed 1 cm above the tip of the lateral malleolus [60–65].

- **Commonly used portals**: Anteromedial and anterolateral.
- **Limitations**: The anteromedial portal may not fully visualize the lateral malleolus.
- **Alternative**: Some surgeons recommend the accessory anterolateral portal and sinus tarsi portal.

Current evidence predominantly relies on observational studies and expert opinions, which constrains the robustness of the conclusions drawn. Future research should prioritize multicenter, prospective randomized controlled trials to assess the impact of different portals on surgical time and clinical outcomes.

### How to address the osteochondral lesions?

#### Recommendation

Bone marrow stimulation is the recommended technique for talar osteochondral lesions (OCL) no more than 150 mm^2^ in area and 5 mm in depth (strength of recommendation: strong; level of evidence: moderate).

#### Statement

When OCL of the talus coexist with CLAI, a combined surgical approach is often required to achieve optimal outcomes. The presence of OCL significantly influences postoperative functional results [66]. Addressing both CLAI and OCL in single-stage surgery demonstrated better short-term clinical outcomes compared to staged procedures [67]. The selection of surgical technique depends on the size and depth of the lesions, regardless of their classification [68,69]. Bone marrow stimulation is considered an ideal procedure for limited OCL. However, there is uncertainty regarding whether a critical defect size exists beyond which bone marrow stimulation may perform poorly. Although Choi *et al.* [70] and Chuckpaiwong *et al.* [71] reported good outcomes for lesions no larger than 150 mm^2^ and 15 mm in diameter following bone marrow stimulation, these guidelines have been reassessed and updated in recent decades. A recent systematic review [72] demonstrated that the optimal lesion size for bone marrow stimulation is less than 107.4 mm^2^ in area and/or 10.2 mm in diameter. OCL smaller than 100 mm^2^ were associated with better American Orthopaedic Foot & Ankle Society (AOFAS) scores compared to patients with OCL larger than 100 mm^2^, with lesion size groups of 100 to 149 mm^2^, 150 to 199 mm^2^, and greater than 200 mm^2^ [73]. For most surgeons, bone marrow stimulation remains the treatment of choice for OCL between 100 mm^2^ and 150 mm^2^ in area and 5 mm in depth, regardless of the lesion’s location, as current evidence has not demonstrated superior outcomes with other treatments for these lesions [74–97]. Recently, autologous matrix-induced chondrogenesis has been applied to the treatment of OCL with promising results, potentially changing the existing limitations regarding the size of osteochondral defects [98–108]. Based on current evidence, we emphasize that the aforementioned size cutoff is recommended, but a more precise threshold should be established as future research updates the findings.

- **Commonly size:** ≤150 mm^2^, ≤100 mm^2^ reported better functional outcomes.
- **Procedure:** Bone marrow stimulation.
- **Alternative**: Autologous matrix-induced chondrogenesis

### One versus two anchors—which is better?

#### Recommendation

The long-term functional outcomes of patients who received one anchor versus two anchors are comparable (strength of recommendation: strong; level of evidence: moderate).

#### Statement

Feng *et al.* [26] compared one suture anchor (*n* = 36) with two suture anchors (*n* = 39) in CLAI patients who underwent arthroscopy. Patients who received one suture anchor had a lower rate of return to sports but achieved comparable AOFAS scores to those who received two suture anchors. Similarly, Li *et al*. [109] compared 20 CLAI patients who underwent a one-anchor procedure with 31 patients who underwent a two-anchor procedure, finding that the two-anchor group had a higher rate of return to sports, with similarly comparable AOFAS scores. Zhou *et al*. [110] performed a one-anchor repair procedure in 22 patients and a two-anchor repair procedure in 24 patients, finding comparable AOFAS, Karlsson ankle function score (KAFS), and time to return to sport between the groups. Similarly, Feng *et al*. [20] found that patients who underwent a one-anchor repair procedure (*n* = 32) achieved similar AOFAS, KAFS, anterior talar translation, and active joint position sense scores at a 24-month follow-up. Although two anchors can provide a larger contact area for the ATFL and facilitate a higher rate of return to sports, both one-anchor and two-anchor repair procedures are suitable for CLAI patients, yielding similar functional outcomes [111–116].

- Number of commonly used anchor: One anchor
- Advantages: Two anchors could provide a larger contact area of the ligament and a higher rate of return to sports

### What is the optimal anchor insertion angle?

#### Recommendation

Positioned parallel to the sagittal plane along the long axis of the fibula and angled at 45° to the coronal plane was recommended (strength of recommendation: expert consensus; level of evidence: not applicable).

#### Statement

Currently, no comparative study has been conducted to examine the differences in anchor insertion angles during ligament repair surgery for CLAI. Notably, recent research has introduced alternative drilling angles in the fibula for ligament reconstruction in the treatment of CLAI. Liu *et al*. [117] drilled 48 fibular tunnels on fresh ankle specimens drilling the bone tunnel at a 60° angle poses a higher risk of fracture from disruption of the lateral fibular cortex. At a 30° angle, the risk of injury to the peroneus longus and brevis tendons is significantly improved (62.5%). In contrast, drilling at 45° reduces the likelihood of injury to the peroneus longus and brevis tendons (31.3%) and the distal fibula, while also providing tunnels of sufficient length. Michels *et al*. [118] recommended creating an oblique fibular tunnel with an angle between 43.7° ± 3.3° and 49.6° ± 10.2° to reduce the risk of distal fibular fractures during reconstruction. Thus, to reduce the risk of fractures during anchor placement and to prevent penetration of the fibula’s double-layered cortex, some researchers recommend inserting the anchor at a 30–45-degree angle to the fibula’s long axis. In a recent study [119], 37 patients with CLAI underwent arthroscopic ATFL repair, with an average follow-up of 33.16 months. The anchor was placed at an angle of 30°–45° relative to the long axis of the fibula. The AOFAS score improved significantly from 73.16 ± 11.23 to 92.53 ± 4.87, while the KAFS increased from 75.02 ± 9.37 to 93.36 ± 6.15. The same group also used suture anchors at the same angles to repair the ATFL in 71 patients with CLAI [120]. Among them, 46 patients returned to their preinjury level of sports, while 25 resumed nonintensive activities. Significant improvements were observed in AOFAS, KAFS, anterior talar translation (ATT), and active joint position sense (AJPS) scores. However, no comparative study has been conducted. Given the risks associated with drilling the fibular tunnel during the procedure, it is recommended to insert the suture anchor parallel to the sagittal plane along the long axis of the fibula, with a 45° angle to the coronal plane.

- Range of the anchor insertion angle: 30°–60°.
- Limitations: A 30° angle carries a high risk of peroneus longus and brevis tendon injuries, while a 60° angle increases fracture risk through lateral fibular cortex disruption.

### What suture configuration should be used?

#### Recommendation

**Figure 2:**
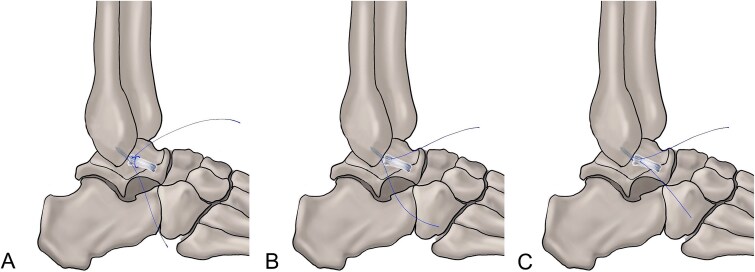
Suture configurations. (A) Loop suture configuration. (B) Free-edge suture configuration. (C) Horizontal mattress suture.

The loop suture configuration, free-edge suture configuration, and horizontal mattress suture configuration are all feasible strategies (Fig. 2) (strength of recommendation: weak; level of evidence: low).

#### Statement

Several studies have investigated whether different suture configurations offer greater benefits for CLAI patients undergoing all-inside arthroscopic repair procedures. The free-edge suture configuration resulted in better KAFS scores and a shorter time to return to full activity compared to the horizontal mattress suture configuration [24]. However, comparable AOFAS scores and ATT values were observed. Additionally, when the loop suture configuration was compared to the free-edge suture configuration, similar AOFAS, KAFS, and AJPS scores were recorded [120]. Takao *et al*. [121] employed a modified lasso-loop suture configuration for CLAI in skeletally immature (*n* = 64) and mature patients (*n* = 103). At 2 years, similar Self-Administered Foot Evaluation Questionnaire scores were reported between the two groups. Liu *et al*. [122] compared the arthroscopic lasso-loop suture configuration (*n* = 32) with the horizontal mattress suture configuration (*n* = 42) in patients with CLAI. At a mean follow-up of 39 months, both groups demonstrated similarly favorable clinical outcomes, including AOFAS, KAFS, and Tegner scores, as well as comparable rates of return to sports and sprain recurrence. Lee *et al*. [123] performed the arthroscopic Broström-Gould procedure with inferior extensor retinaculum (IER) augmentation using a lasso-loop suture configuration, facilitated by a knot pusher and a semiconstrained freehand tie in 135 patients with CLAI. The procedure resulted in significant improvements in both AOFAS scores and 12-Item Short Form Survey outcomes. Guo *et al*. [124] and Qin *et al.* [125] produced similar results, further validating the effectiveness of the lasso-loop suture configuration. Liu *et al.* [126] compared the modified Mason–Allen suture with the horizontal mattress suture in 64 patients and demonstrated comparable functional outcomes at intermediate follow-up.

- **Commonly used suture configurations**: Loop and free-edge suture configurations.
- **Alternative**: Horizontal mattress suture configuration.

### Is it necessary to preserve the remnant when performing the anatomic reconstruction?

#### Recommendation

**Figure 3:**
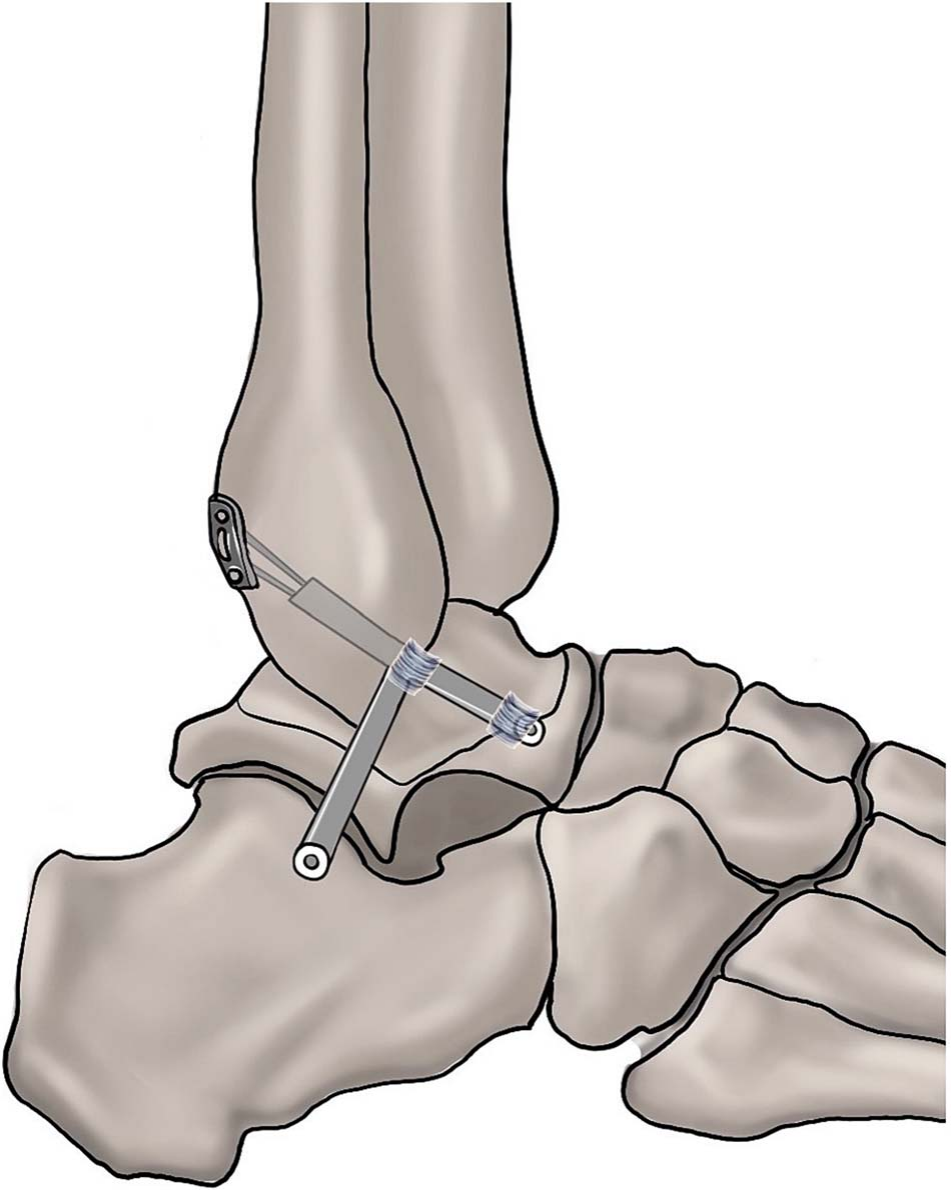
Ligament remnant preservation reconstruction.

Remnant preservation is not necessary (Fig. 3) (strength of recommendation: strong; level of evidence: moderate).

#### Statement

A randomized controlled study assessed the necessity of remnant preservation during arthroscopic reconstruction [127]. The study included two groups: the preservation group (*n* = 25), where the remnant of the ATFL on the fibula was preserved, and the nonpreservation group (*n* = 28), where the ATFL remnant was not preserved. After a follow-up period of more than 30 months, the AOFAS score, anterior talar translation, KAFS, and active joint position sense were comparable between the two groups, indicating that remnant preservation was not necessary during the arthroscopic reconstruction procedure. A total of 182 consecutive patients across two centers underwent ATFL/CFL reconstruction without remnant preservation [128]. At a mean follow-up of 23 months, the AOFAS and KAFS scores improved to 86.5 ± 18.7 and 85 ± 18.3, respectively. Lan *et al*. [25] reported the outcomes of all-inside arthroscopic ATFL reconstruction without remnant preservation in 15 high-demand patients. After a mean follow-up of 19.5 ± 1.8 months, the anterior talar translation improved from 13.2 ± 1.5 mm to 4.8 ± 1.1 mm, and the AOFAS score increased from 56.8 ± 10.5 to 90.2 ± 6.2. Zhang *et al*. [11] preserved the ATFL remnant during the reconstruction procedure in 28 patients, resulting in an improvement in AOFAS scores from 63.3 ± 6.9 to 91.9 ± 6.8 and KAFS scores from 55.2 ± 6.9 to 95.3 ± 6.7. Dong *et al*. [129] performed remnant preservation reconstruction in 20 patients, with a significant improvement in AOFAS scores 12 months after surgery (79.7 ± 4.3) compared to preoperative scores (52.0 ± 4.1). Ligament reconstruction without remnant preservation can effectively restore ankle function with a low risk of complications [130–135].

- **Commonly used procedures:** Ligament reconstruction without remnant preservation.
- **Advantages**: The nonremnant preservation approach demonstrates lower complication risks.

### How to deal with unstable os subfibulare?

#### Recommendation

**Figure 4:**
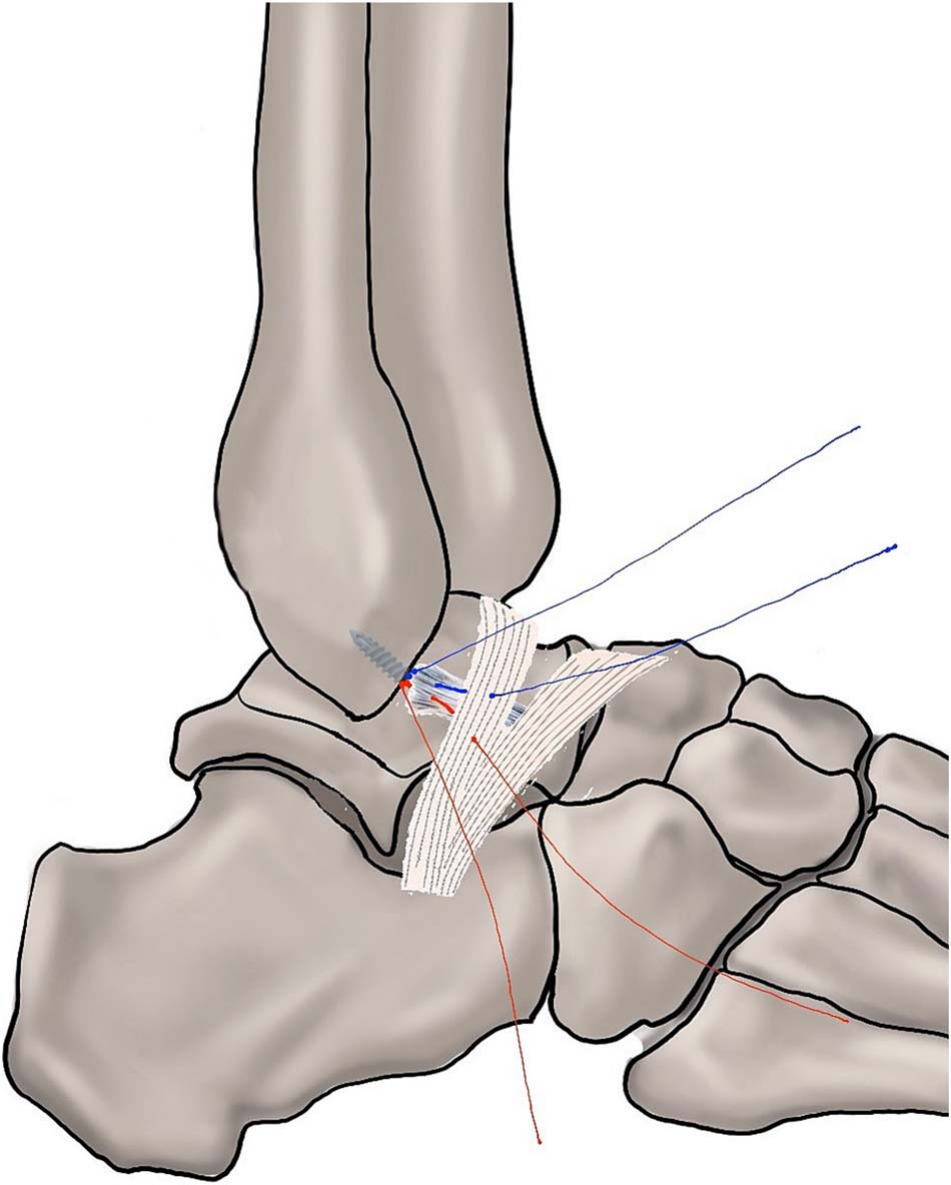
Repair of the remnant of the ligament with augmentation (Broström–Gould).

Arthroscopic excision and repair of the remnant of the ligament with augmentation are the recommended procedure (Fig. 4) (strength of recommendation: strong; level of evidence: moderate).

#### Statement

Although fixation of the unstable os subfibulare to the fibula is feasible [136–138], excision of the os subfibulare remains the most commonly used approach [139–143]. When the volume of the remnant ATFL is greater than half of the normal ligament, repair procedures are performed [144]. Kubo *et al*. [145] performed ossicle resection and lateral ligament repair in 31 adolescent patients, with improved AOFAS and KAFS scores. The average time for patients to return to school physical education after surgery was 11.4 ± 1.6 weeks. However, if the remaining ATFL volume is less than half, reconstruction procedures are employed [136,142,146,147]. Cao *et al*. [148] conducted a study on 16 patients with CLAI and accessory ossicles. After ligament reconstruction, KAFS improved from 52.7 ± 15.1 to 86.4 ± 8.2. Additionally, the varus talar tilt angle decreased from 15.4 ± 2.0° to 6.2 ± 1.6°, and anterior talar displacement reduced from 14.3 ± 2.1 mm to 6.3 ± 1.4 mm. Patient satisfaction was reported at 87.5%. In the future, controlled studies comparing repair and reconstruction techniques for CLAI patients with an os subfibulare will be essential to determine the optimal surgical approach.

- **Commonly used procedures**: Excision of the os subfibulare and repair the ligament
- **Alternative**: Fixation of the unstable os subfibulare.

### Anatomic reconstruction, Broström or Broström–Gould repair—which is better?

#### Recommendation

**Figure 5:**
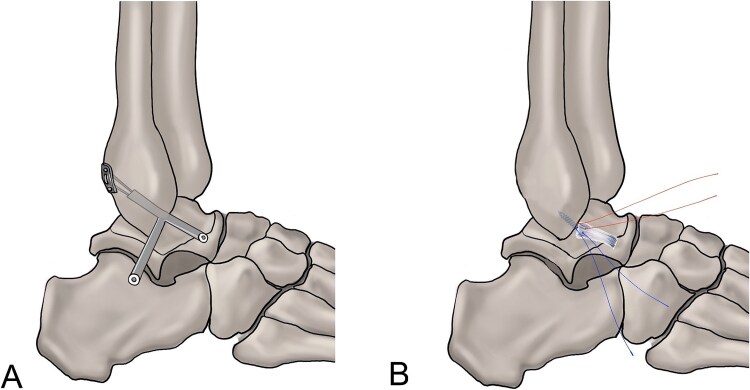
(A) Anatomic reconstruction procedure. (B) Broström repair procedure.

Arthroscopic repair procedures are the gold standard for the management of CLAI (Figs 4 and 5) (strength of recommendation: strong; level of evidence: high).

#### Statement

Despite significantly lower anterior talar translation and talar tilt angle observed in the arthroscopic ligament reconstruction procedure, the clinical outcomes (VAS, AOFAS, KAFS) were comparable to those of the Broström–Gould procedure [149]. However, the arthroscopic reconstruction approach was associated with a longer recovery time [150]. Based on the six articles included in a meta-analysis [151], with an average minimum follow-up of 29.2 months, arthroscopic repair demonstrated superior clinical outcomes (KAFS, AOFAS, and Tegner score), lower complication rates, and faster return to pre-injury sports, supporting its use as the gold standard for the treatment of CLAI [150,152–155]. The arthroscopic repair procedure yielded good outcomes for CLAI patients [156–163], resulting in an intact ATFL with normal morphology [154,164]. Even for athletes, anatomic repair remains the preferred technique for primary surgery [165]. Anatomic graft reconstruction can replicate the angular stability of the native ligament in cadaver models [166], but the clinical outcomes remain comparable to the repair procedures [167]. For patients with poor-quality or absent ATFL remnants, where reconstruction procedures are typically required, ATFL repair alone has not been sufficient to achieve favorable results [168,169]. However, arthroscopic modified Broström–Gould repair has shown functional outcomes comparable to those of open ATFL repair with augmentation using the IER [23,29]. Arthroscopic repair and reconstruction procedures to manage CLAI offer high patient satisfaction in the midterm, with durable results and a low rate of complications [22,25,27,170–174].

- **Commonly used procedures**: Repair (Broström or Broström–Gould)
- **Advantages:** Fast recovery without tissue graft.
- **Limitations:** Repair alone is not sufficient to patients with poor-quality or absent ATFL remnants
- **Alternative**: Reconstruction.

### When to start range of motion and weight-bearing?

#### Recommendation

Early range of motion exercises for the knee and toes are recommended to prevent joint stiffness. The operated ankle should be immobilized with a brace for the first 2 weeks without weight-bearing. During the following 4 weeks, full weight-bearing is allowed using the walking boot. For patients who underwent bone marrow stimulation, weight-bearing was not recommended during the first 6 weeks after surgery (strength of recommendation: expert consensus; level of evidence: not applicable).

#### Statement

No study to date has specifically addressed postoperative care following arthroscopic treatment for CLAI. Although early weight-bearing helps prevent muscle atrophy and promotes functional recovery, immobilization for 2–4 weeks post-surgery is essential to support optimal healing of the repaired tissue [175,176]. On the second day following surgery, early active functional exercises for the operated limb can be initiated, including nonweight-bearing movements and isometric exercises [177,178]. Static and dynamic balance exercises can enhance both ankle strength and dynamic stability [179–185]. The walking boot can be used to support early full weight-bearing for 2–4 weeks following a two-week period of immobilization. Afterward, the boot is removed, and patients are allowed to walk fully weight-bearing starting at 7–8 weeks [186,187]. For CLAI patients with associated OCL who undergo bone marrow stimulation, although early weight -bearing produced good functional outcomes [188–191], non-weight-bearing should be limited to no more than 6 weeks postsurgery [192–195].

- **Time to start range of motion:** Immediately after the surgery.
- **Time to weight-bearing:** 2 weeks after surgery.

### When to return to work and sports?

#### Recommendation

Resumption of work should occur no earlier than 6 weeks post-surgery, while recreational sports activities should not begin before 8 weeks. For patients with OCL, a delay of at least 6 weeks beyond these timelines is recommended (strength of recommendation: expert consensus; level of evidence: not applicable).

**Table 2:** Evidence-based recommendations for all-inside arthroscopic CLAI management

Recommendation	Strength of recommendation	Level of evidence
All-inside arthroscopic management is indicated when (i) Patients experience functional impairments (such as pain, recurrent ankle sprains, or giving way) despite more than 6 months of conservative management; (ii) A positive anterior drawer test or talar tilt test, along with imaging (MRI, stress radiography or stress ultrasound) confirms CLAI	Expert consensus	Not applicable
The anteromedial, anterolateral, accessory anterolateral, and sinus tarsi portals are the most commonly used approaches	Expert consensus	Not applicable
Bone marrow stimulation is the recommended technique for talar OCL no more than 150 mm^2^ in area and 5 mm in depth	Strong	Moderate
The long-term functional outcomes of patients who received one anchor versus two anchors are comparable	Strong	Moderate
Positioned parallel to the sagittal plane along the long axis of the fibula and angled at 45° to the coronal plane was recommended	Expert consensus	Not applicable
The loop suture configuration, free-edge suture configuration, and horizontal mattress suture configuration are all feasible strategies	Weak	Low
Remnant preservation is not necessary	Strong	Moderate
Arthroscopic excision and repair the remnant of the ligament with augmentation are the recommended procedure	Strong	Moderate
Arthroscopic repair procedures are the gold standard for the management of CLAI	Strong	High
Early range of motion exercises for the knee and toes are recommended to prevent joint stiffness. The operated ankle should be immobilized with a brace for the first 2 weeks without weight-bearing. During the following 4 weeks, full weight-bearing is allowed using the walking boot. For patients who underwent bone marrow stimulation, weight-bearing was not recommended during the first 6 weeks after surgery	Expert consensus	Not applicable
Resumption of work should occur no earlier than 6 weeks post-surgery, while recreational sports activities should not begin before 8 weeks. For patients with OCL, a delay of at least 6 weeks beyond these timelines is recommended	Expert consensus	Not applicable

#### Statement

Bouveau *et al*. [196] studied 40 patients with CLAI who underwent arthroscopic repair or reconstruction procedures. Of these 40 patients, 30 successfully resumed sports activities, achieving this milestone at an average of 6.0 months. Notably, patients with strong preoperative motivation returned to sports in an average of 4.5 months. Teramoto *et al*. [197] performed an arthroscopic repair procedure followed by accelerated rehabilitation in 20 patients, with 75% returning to sport 8 weeks postoperatively. Early weight-bearing may facilitate a quicker return to sports activities for patients postoperatively [198]. Liu *et al*. [122] reported return to work at 10 weeks and return to sports at 18 weeks for 74 patients with CLAI who underwent arthroscopic repair procedures. Cordier *et al*. [27] reported a mean time of 3 months (range 0.5–7 months) for return to work among 53 patients who underwent an arthroscopic reconstruction procedure. A recent meta-analysis [199], which included data from 25 studies involving 1384 participants, found that the average time to return to sports was 12.45 weeks (10.8–14.1 weeks). Compared to open surgery, patients who underwent arthroscopic surgery were able to return to sports within 8–18 weeks [23,24,200–202]. However, patients with cartilage damage experienced delayed recovery, with return to work and sports typically delayed by 2–4 months compared to those with isolated CLAI [203–205]. For athletes, training should not begin earlier than 3 months after surgery, with sports-specific training starting at 6 months post-surgery [206]. A recent meta-analysis, which included data from 227 studies, recommends that return to nonspecified impact sports should occur no earlier than 12 weeks [192].

- **Time to return to work**: 6 weeks after the surgery.
- **Time to return to sport**: 8–18 weeks after the surgery.
- **Advantage**: Early weight-bearing may facilitate a quicker recovery.

## Conclusion

The clinical practice guidelines for all-Inside arthroscopic surgery for CLAI are the first evidence-based guidelines developed in this field. These guidelines (Table 2) aim to provide recommendations for orthopedic surgeons, with the goal of improving the quality of care for patients undergoing all-inside arthroscopic surgery for this condition. However, given the limited availability of high-quality evidence, many of the recommendations rely primarily on expert consensus, resulting in guidelines of moderate strength. In patients with CLAI undergoing all-inside arthroscopic procedures, comparative studies are urgently needed to establish the optimal timing for weight-bearing, as well as return to work and sports. Further multicenter randomized controlled trials are necessary to refine these recommendations and improve CLAI management.

## Data Availability

No new data were generated or analyzed for this research.
